# Repeatability and test–retest reliability of thermal and pressure pain threshold testing in healthy thoroughbred horses

**DOI:** 10.3389/fvets.2026.1887508

**Published:** 2026-07-03

**Authors:** J. L. Khatib, C. De Gennaro, T. M. Masters, S. L. Messer, R. A. Moura, M. F. Mallicote, L. Chiavaccini

**Affiliations:** 1 Department of Comparative, Diagnostic and Population Medicine, College of Veterinary Medicine, University of Florida, Gainesville, FL, United States; 2 Department of Large Animal Clinical Sciences, College of Veterinary Medicine, University of Florida, Gainesville, FL, United States

**Keywords:** equine, pain pressure threshold, quantitative sensory testing, repeatability, test–retest reliability, thermal threshold

## Abstract

**Introduction:**

Quantitative sensory testing (QST) has been used in horses to assess nociceptive responsiveness but its broader clinical application is limited by variability across anatomical sites, environmental conditions, operator technique, and behavioral endpoints. This study aimed to evaluate the feasibility, within-session repeatability, and test–retest reliability of thermal threshold (TT) and pressure pain threshold (PPT) testing in healthy Thoroughbred horses.

**Methods:**

Eleven adult Thoroughbred horses (4 mares, 7 geldings; median [IQR] age 6 [5–6] years; body weight 558 ± 47 kg) underwent QST on two occasions 1 week apart. Thermal threshold testing was performed at the nostrils, withers, and coronary band using a handheld contact heat probe (0.6 °C/s ramp; 55 °C cut-off). Pressure pain threshold testing was assessed at the temporomandibular joint (TMJ), seventh rib, and dorsal metacarpophalangeal joint (D-MCP) using a blunt-tipped pressure algometer (2,500 g safety cut-off). Three repeated measurements were collected at each site per session. Within-session repeatability was assessed by coefficient of variation (CV) and test–retest reliability by intraclass correlation coefficient (ICC), calculated from the average of the two closest of three measurements per session.

**Results:**

TT showed moderate to high within-session repeatability (CV 8.9%–13.6%) but poor test–retest reliability at all sites. PPT within-session repeatability was low (CV 34%–57%), with test–retest reliability varying by site: the D-MCP showed moderate reliability, the TMJ fair, and the seventh rib poor.

**Discussion:**

Equine QST reliability is strongly influenced by site selection, environmental conditions, behavioral responsiveness, the biological homogeneity of the cohort, which restricted between-subject variance. These findings support QST as a feasible research tool in horses but highlight the need for repeated trials, strict temperature control, and consistent operator technique. Validation in larger, more diverse populations and in horses with naturally occurring pain conditions is warranted.

## 1 Introduction

Quantitative sensory testing, a structured psychophysical approach to measuring sensory and nociceptive thresholds has been adapted for use in horses to investigate analgesia and quantify nociceptive responsiveness in different scenarios (1–6). Two modalities are commonly used in equine clinical research: contact heat thermal threshold (TT) and pressure pain threshold (PPT) testing. Equine validation work has shown that mechanical, electrical, and thermal nociceptive tests can be applied safely, but that performance depends on careful standardization and clear behavioral endpoints (7, 8). Thermal threshold can be influenced by tissue thickness, local perfusion, probe contact, probe heating ramp, and environmental or skin temperature, while PPT measurements are sensitive to operator probe placement/technique and rate of force application (7, 9). More recent equine studies applying QST to specific body regions, have made evident that threshold values and reliability can be strongly site-dependent (10–12). In general, skin overlying bony surfaces showed higher mechanical nociceptive thresholds than soft tissue-covered areas, likely reflecting differences in stimulus dissipation or mechanoreceptor density in this species (11). Moreover, environmental conditions, environmental distractors and a learning component may affect the response to nociceptive testing (9, 10). Because of this variability, QST is uncommonly used for objective measurement of nociception (or absence thereof) in the clinical settings.

However, due to growing support for QST as a valid, semi-objective component of comprehensive pain evaluation in humans and the canine species (13–16), the development of similar approaches is warranted in equine medicine. A validated, reliable, and quantitative method to assess somatosensory function in horses is essential to characterize the extent and nature of peripheral and central sensitization, and thus to guide adequate and individualized pain management. Preliminary work suggested that QST could be implemented for example for evaluating equine trigeminal sensory function and for characterization of the patient-specific osteoarthritic pain phenotype, however with reliability that varied by anatomical site and testing conditions (11, 12). Several trials explored the usability of QST to evaluate back pain (17) or alteration of limb sensitivity (18) or trigeminal sensation (11, 19) in equines, and summarizing reviews of results obtained with these method in horses have been published (7, 9). Before TT and PPT are used to compare individuals or detect change over time, it is essential to quantify both (i) repeatability within a single testing session and (ii) test–retest reliability across sessions (how stable measurements are between days).

The aim of the present study was to assess the feasibility, intra- and inter-session repeatability of (a) PPT using a blunt-probed pressure algometer and (b) TT using a thermode-based system, at six different body sites previously used in equine clinical research. We hypothesized that both TT and PPT would be feasible in healthy horses and that threshold measurements would demonstrate acceptable within-session repeatability, with the PPT at the dorsal metacarpophalangeal joint (D-MCP) and TT at the withers showing superior test–retest reliability compared to other sites.

## 2 Materials and methods

### 2.1 Ethical statement

This single-center, randomized, Latin-square experimental study was conducted in compliance with institutional ethical standards and approved by the University of Florida Institutional Animal Care and Use Committee (IACUC 202500000148 and IACUC 202500000169) and College of Veterinary Medicine Hospital Research Review Committee (VHRRC) at the University of Florida, FL, USA.

### 2.2 Animals

Eleven adult Thoroughbred horses (four mares and seven geldings) from the University of Florida Research Herd were enrolled as part of two concomitant studies. Age ranged from 4 to 17 years, with a median [interquartile range (IQR)] of 6 (5, 6) years, and mean ± SD body weight was 558 ± 47 kg. All horses were considered healthy based on history, routine physical examinations, complete blood count, and serum biochemistry and they were free of manifest chronic lameness. Under standard housing conditions, horses were kept in pastures in groups of two to four. On each study day, horses were removed from the pasture in pairs, weighed, and transferred to adjacent individual stalls (4 × 4 m) in the research barn. A physical examination was performed upon arrival to confirm health status prior to testing. Horses were allowed a minimum of 1 hour of acclimatization before data collection began. Throughout the observation period, horses had visual and auditory contact with their companion through the open upper bars of the stall, received hay at regular intervals, and had access to water ad libitum. When necessary horses were sprayed with an insect repellent. At the end of each session, horses were returned to their herd.

### 2.3 Study design

**Figure 1:**
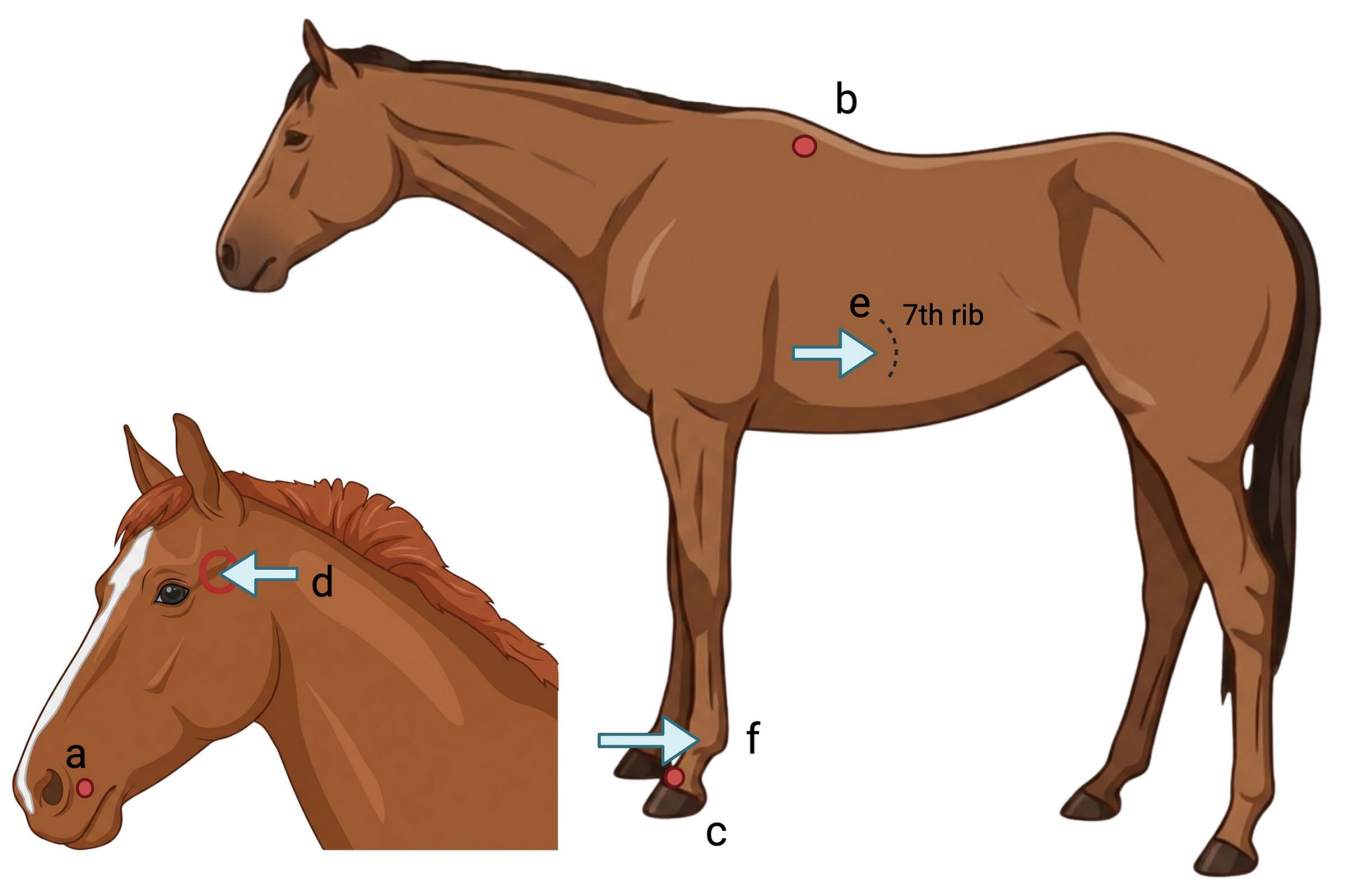
Schematic representation of the testing sites. The thermal threshold probe was tested at three different anatomic sites (red dot): the nostril **(a)**, the withers **(b)**, and the coronary band **(c)**. Pressure pain threshold (arrow) was assessed at the temporomandibular joint **(d)**, the 7th rib **(e)**, and the dorsolateral aspect of the metacarpal-phalangeal joint **(f)**, using a blunt-tipped pressure algometer. Image created using BioRender.com.

After physical examination, each horse was loosely manually restrained using a halter and a lead rope for TT and PPT sensory testing. Three repeated measurements 20 min apart were performed under each stimulation condition. The hair over five body sites on the left side of the horse were clipped for testing: the withers, the coronary band on the dorsal aspect of the pastern, the temporomandibular joint (TMJ) as described by Veres-Nyéki et al. (11), the 7th rib at the costochondral junction, and the D-MCP as described by Gisler et al. (12). The nostrils were not shaved (Figure 1). The TT was always performed before the PPT, but the testing order of different sites was randomized using open-source software^1^. The procedure was repeated after 1-week washout period.

#### 2.3.1 Thermal threshold (TT)

Thermal thresholds were measured using a handheld contact heat probe with a smooth flat tip (NTE-3A, Physitemp Instruments, Clifton, NJ, USA; 13 mm contact surface) attached to a 10-foot flexible lead, a heating ramp of 0.6 °C/s, and a safety cut-off of 55 °C. The probe incorporates a Peltier semiconductor heat pump and a digital temperature control unit to ensure accurate temperature delivery, as previously described (20). Three anatomical sites were tested: the nostril, the withers, and the coronary band (Figure 1). Skin temperature at each site was recorded before stimulation using a commercially available infrared thermometer laser temperature gun (Etekcity Corp., Anaheim, CA, USA) and the probe baseline temperature was set to 32 °C. The probe was then positioned on the target site without applied pressure and held at baseline temperature for approximately 20–30 s to allow the horse to habituate to contact (defined as arrest of the panniculus muscle twitching) and inhibiting mechanoception. Heating was subsequently initiated by a second operator, not involved in behavioral observation, using the remote control on the NTE-3 monitor. Probe temperature increased at 0.6 °C/s until a behavioral response was observed, defined as panniculus muscle twitching, a leg lift, lateral body displacement or evasion or turning the head toward the probe, or until the safety cut-off of 55 °C was reached. Testing was terminated remotely by the operator, who was unaware of the temperature reached at the time of response. Trials in which no response occurred before the cut-off were recorded at 55 °C, consistent with established equine TT testing practice (8). Given the potential confounding effect of cold ambient conditions on thermal sensory conduction, and the tendency of the coronary band to equilibrate rapidly to hoof and floor temperature, any site with a recorded skin temperature below 25 °C was warmed to 30 °C–32 °C prior to testing.

#### 2.3.2 Pressure pain threshold (PPT)

Pressure pain threshold were measured using a handheld pressure algometer (SMALGO, Bioseb, Vitrolles, France) fitted with a flat 3-mm diameter tip connected to a digital recording unit sampling at 1000 Hz, with an accuracy of 0.2% and a resolution of 0.1 g. Three anatomical sites were assessed: the TMJ, the 7th rib, and the D-MCP (Figure 1). The device was calibrated before each recording, following manufacturer instructions. The probe was positioned perpendicular to the skin surface at each site, and force was increased steadily at a target rate of approximately 200 g/s (or approximately 3 kg/cm^2^/s) until a clear avoidance response was observed, as previously defined, or until the safety cut-off of 2,500 g was reached. The threshold was recorded automatically upon disconnection of the probe, at which point data capture ceased. The operator had visual access to the digital display during testing, but was unaware of the pressure until the stimulation was aborted.

All QST measurements were performed by the same operator throughout the study.

### 2.4 Statistical analysis

Based on recent results reported by Gisler et al. (12), assuming an ICC *ρ* = 0.68 with an expected width of the 95% confidence interval (CI) of 0.3, two values per subject and a drop-out rate of 20%, nine horses were considered necessary to assess test–retest reliability. A total of 11 horses were enrolled.

Data elaboration and analysis were performed with Stata/BE 17.0 for Mac (StataCorp LLC, College Station, TX) and SigmaPlot 14 (Systat Software, Palo Alto, CA). Continuous data were checked for normality of distribution using the Shapiro–Wilk normality test and graphically with histogram and the normal quantile plot function in Stata. Data are reported as mean ± SD and median (IQR) if normally or not normally distributed, respectively. Categorical data are presented as proportions or percentages. Descriptive statistics was used for demographic data. Environmental temperature between sessions was compared using two-sample t test with unequal variances.

Repeatability was calculated using the coefficient of variation (CV) between three measurement taken on the same session and defined as follows: CV < 10%: high repeatability; 10% ≤ CV < 20% moderate repeatability; CV ≥ 20%: low repeatability) as previously described (20). Test–retest reliability (across two sessions) was assessed using a two-way mixed-effects ICC model for absolute agreement, calculated on the average of the two closest of three repeated measurements per session, as previously proposed (7, 12, 13). The ICC values were classified as follows: <0.20 poor agreement; 0.21–0.40 fair agreement; 0.41–0.60 moderate agreement; 0.61–0.80 good agreement and >0.80 excellent agreement as previously recommended (12). Bland–Altman plots were performed to graphically represent differences between TT and PPT between two sessions. A mixed-effect linear model was fit for TT and PPT, with horse as random effect, sex, testing site, session and their interaction as fixed effect, followed by postestimation and analysis of normality distribution of residuals. *p*-values ≤ 0.05 were considered statistically significant.

## 3 Results

All horses were familiar with the testing device and did not tried to avoid testing. No burns or visible skin trauma (erythema, oedema, or breach of skin integrity) were detected at any stimulus site at any time during the study.

### 3.1 Thermal threshold

Mean ± SD ambient temperature during data collection was 14 °C± 3 °C in the first session and 11 °C± 7 °C in the second, with no significant difference between sessions (mean difference 3 °C, SE 2 °C, 95% CI − 3 to 9 °C; t = 1.11, *p =* 0.30). Aversion response rates were 100% at the nostrils, 94% (62/66 measurements) at the withers, and 84% (55/66 measurements) at the coronary band.

**Figure 2:**
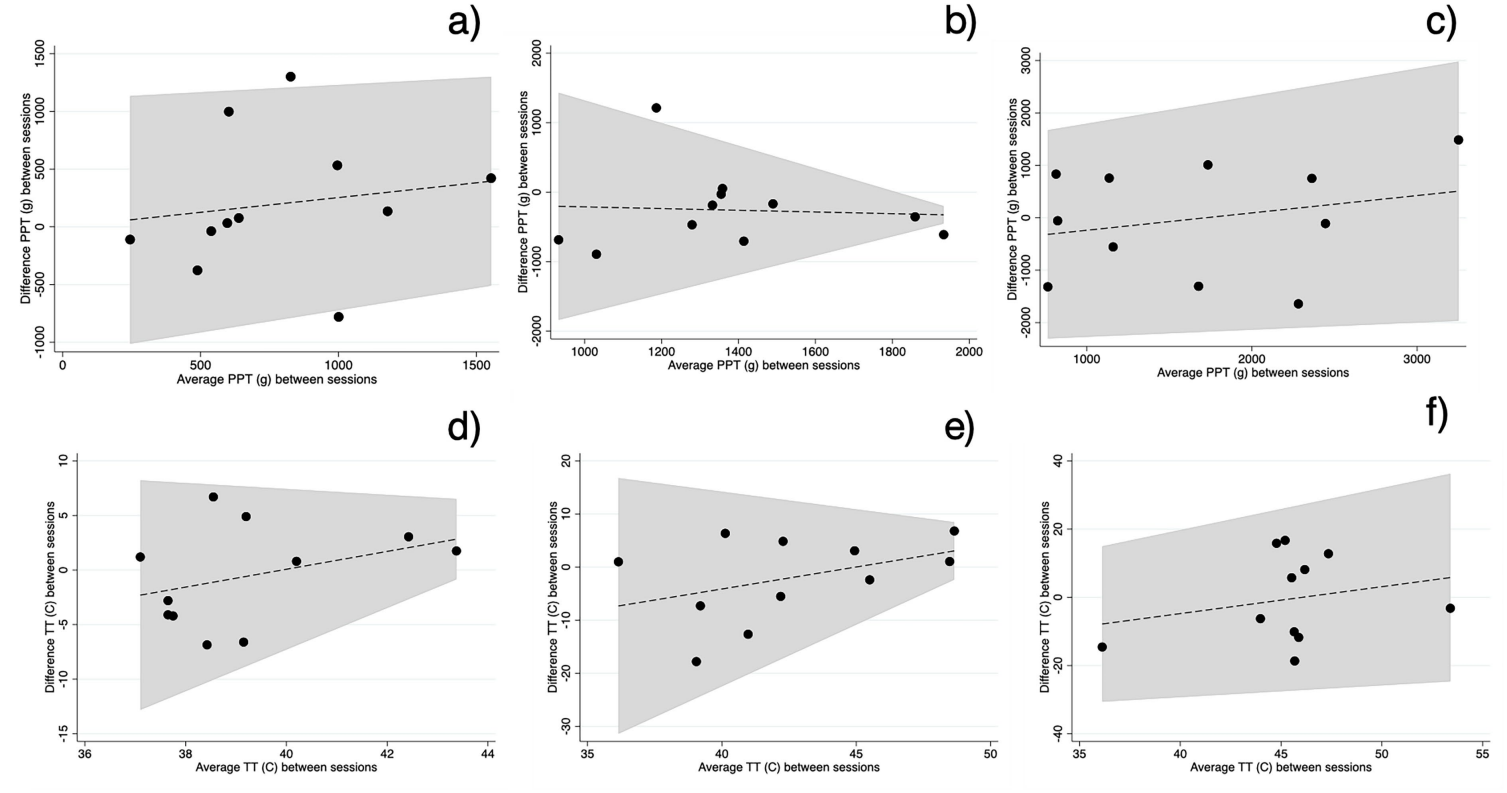
Bland–Altman plots illustrating agreement between sessions for mechanical and thermal thresholds at each anatomical site in 11 Thoroughbred horses. Panels **(a–c)** show pressure pain threshold (PPT) measurements recorded between the two sessions at the temporomandibular joint (TMJ), 7th rib, and dorsal metacarpophalangeal (D-MCP) joint, respectively. Panels **(d–f)** show thermal threshold (TT) measurements recorded between the two sessions at the nostrils, withers, and coronary band, respectively. The grey band represents the 95% confidence interval. At the 7th rib **(b)** and the D-MCP **(c)**, the funnel-shaped confidence band widening toward higher average values suggests proportional bias, as measurement error increases as thresholds increase. At the nostrils **(d)** and the withers **(e)**, differences cluster reasonably close to zero with a relatively narrow band.

During the first session, mean ± SD TT across three repeated measurements was 39.5 °C± 4.5 °C at the nostrils, 43.6 °C± 5.9 °C at the withers, and 46.2 °C± 6.6 °C at the coronary band. Within-session repeatability ranged from moderate to high, with mean ± SD CV of 8.9% ± 4.0% at the nostrils, 12.7% ± 5.0% at the withers, and 13.6% ± 5.0% at the coronary band. For further analysis, the two closest of three measurements were averaged per session; the resulting values are reported in Table 1. Between-session reliability was poor at all sites: ICC was −0.36 (95% CI − 1.67 to 0.73; *p =* 0.65) at the nostrils, −0.09 (95% CI − 4.69 to 0.73; *p* = 0.55) at the withers, and −1.03 (95% CI − 33.99 to 0.34; *p* = 0.92) at the coronary band. Non-significant F-tests at all sites confirmed the absence of a systematic directional change in thresholds between sessions. Test–retest reliability is summarized in Table 2 and Bland–Altman plots illustrating between-session agreement at each site are presented in Figure 2.

**Table 1:** Mean ± SD of the two closest of the three values recorded in 11 Thoroughbred horses at three body sites, recorded during two sessions 1 week apart.

Site	Session	
	I	II
Thermal threshold		
Nostrils	38.9 °C± 2.7 °C	39.6 °C± 3.4 °C
Withers	42.4 °C± 4.9 °C	42.6 °C± 6.5 °C
Coronary band	44.6 °C± 6.5 °C	46.3 °C± 8.3 °C
Pressure pain threshold		
TMJ	700.11 ± 450.02 g	874.95 ± 501.48 g
7th rib	1512.95 ± 429.04 g	1245.45 ± 402.43 g
D-MCP	1565.36 ± 852.50 g	1789.11 ± 1083.94 g

D-MCP, dorsal metacarpophalangeal joint; TMJ, temporomandibular joint.

**Table 2:** Test re-test reliability across sessions.

Site	ICC	95% CI	Interpretation
Thermal threshold			
Nostrils	−0.13	−0.74 to 0.50	Poor
Withers	−0.04	−0.70 to 0.57	Poor
Coronary band	−0.49	−0.94 to 0.20	Poor
Pressure pain threshold			
TMJ	0.34	−1.40 to 0.82	Fair
7th rib	0.11	−1.54 to 0.74	Poor
D-MCP	0.60	−0.49 to 0.95	Moderate

Two-way mixed-effects ICC for absolute agreement. Values based on the average of the two closest of three repeated measurements per session. D-MCP, dorsal metacarpophalangeal joint; TMJ, temporomandibular joint.

On one recording day, ambient temperature was markedly lower (2.1 °C), potentially influencing TT responses despite pre-warming of test sites. After excluding the two horses tested under these conditions (*n* = 9), between-session reliability remained poor at the nostrils (ICC = 0.00, 95% CI − 0.74 to 0.66) and the coronary band (ICC = −0.77, 95% CI − 1.10 to 0.03), while the withers showed marginal improvement to fair reliability (ICC = 0.34, 95% CI − 0.23 to 0.78).

In the mixed-effects model examining the association between TT and sex, testing site, session, and their interaction, the overall model was statistically significant (Wald *χ*^2^ = 19.8, *p* < 0.01). After adjustment for all covariates, TT did not differ significantly between sessions (coefficient = 1.66, SE = 2.26, *z* = 0.73, 95% CI − 2.77 to 6.09; *p* = 0.46), and no day-by-site interaction was detected (LR *χ*^2^ = 0.20, *p* = 0.90). The threshold reached was significantly lower at the nostrils than at the coronary band (coefficient = −5.72, SE = 2.26, *z* = −2.53, 95% CI − 10.15 to −1.28; *p* = 0.01). Geldings had significantly higher TT than mares (coefficient = 2.74, SE = 1.36, *z* = 2.02, 95% CI 0.08 to 5.40; *p* = 0.04).

### 3.2 Pressure pain threshold

During the first session, median (IQR) PPT across three repeated measurements was 771 (395–1,389) g at the TMJ, 1479 (1051–1,689) g at the 7th rib, and 1,495 (992–2,352) g at the D-MCP joint. Within-session repeatability was low across all sites, with mean ± SD CV of 57% ± 28% at the TMJ, 36% ± 15% at the 7th rib, and 34% ± 17% at the D-MCP. The two closest of three measurements were averaged per session for further analysis; the resulting values are reported in Table 1. Between-session reliability ranged from poor to moderate: ICC was 0.34 (95% CI − 1.40 to 0.82; *p* = 0.26) at the TMJ, 0.11 (95% CI − 1.54 to 0.74; *p* = 0.42) at the 7th rib, and 0.60 (95% CI − 0.49 to 0.95; *p* = 0.09) at the D-MCP joint. Non-significant F-tests confirmed the absence of a systematic change in thresholds between sessions. Test–retest reliability is summarized in Table 2 and Bland–Altman plots are presented in Figure 2.

In the mixed-effects model examining the association between PPT and sex, testing site, session, and their interaction, the overall model fitted the data well (Wald *χ*^2^ = 31.9, *p* < 0.001). After adjustment for all covariates, PPT did not differ significantly between mares and geldings (coefficient = −127.16, SE = 239.16, *z* = −0.53, 95% CI − 595.91 to 341.58; *p* = 0.59), between sessions (coefficient = −267.50, SE = 238.18, *z* = −1.12, 95% CI − 734.32 to 199.33; *p* = 0.26), or across the day-by-site interaction (LR *χ*^2^ = 2.52, *p* = 0.28). PPT was significantly lower at the TMJ than at the 7th rib (coefficient = −591.67, SE = 172.33, *z* = −3.43, 95% CI − 929.43 to −253.92; *p* = 0.001).

## 4 Discussion

In this cohort of healthy Thoroughbred horses, TT demonstrated moderate to good within-session repeatability, whereas test–retest reliability across 1 week was poor at all sites. By contrast, PPT within-session repeatability was low across all sites, and test re-test reliability was variable among sites, with D-MCP yielding the highest ICC among PPT sites. These findings partially supported our hypothesis: within-session repeatability was higher for TT than PPT, while between-session reliability was limited for both modalities in this homogeneous cohort.

Low within-session CVs for TT indicate that repeated trials clustered closely when administered 20 min apart, consistent with observations in laboratory animals where thermal thresholds remained stable over a 5-h period when inter-trial intervals exceeded 15 min (21). However, near zero or negative ICCs values indicate that within-subject variability across days exceeded between-subject variability, preventing reliable discrimination of individual horses across sessions. This pattern is expected in a homogeneous cohort where between-horse differences are inherently small relative to day-to-day measurement variability, a phenomenon previously reported in QST studies in other species (13, 20), and was corroborated in the present study by the absence of a statistically significant association between TT and testing session. This characteristic has direct clinical implications. In experimental contexts where the outcome of interest is a group mean response, such as pharmacological studies evaluating antinociceptive drug effects, low test–retest ICC does not meaningfully compromise the validity of the approach. Conversely, the ability to detect clinically meaningful changes in TT within a single animal over time needs to be better studied in a more heterogenous equine cohort. Consistent with previous equine studies, threshold values differed significantly across anatomical sites (10, 11). Interestingly, recorded TT was significantly higher in geldings than in mares. Sex effects on nociceptive thresholds remain inconclusive in the equine literature (9). Veres-Nyéki et al. (11) found that sex does not affect facial QST thresholds, while sex-dependent differences have been reported in humans (22), dogs (23) and rodents (24). Some authors have suggested that inclusion of both mares and geldings may contribute to threshold variability (25), and have recommended restricting testing to mares during diestrus to minimize hormonal confounding (26). The finding in this study is incidental, and the study was not powered to detect it, so these results should be interpreted with caution. An uneven distribution of genders and the absence of data on the estrous cycle status of the four mares represent limitations of this study and could have impacted the results. Future studies specifically addressing sex differences in thresholds are therefore warranted. Until better knowledge is acquired, a well-balanced study population with respect to sex is therefore recommended when TT is used in research.

Pressure pain threshold showed considerably greater within-session variability than TT. Among the sites assessed, D-MCP demonstrated the highest between-session ICC, consistent with periarticular mapping studies in which PPT at distal limb landmarks yielded good repeatability and intra- and inter-rater reliability (12). The observed variability supports using repeated trials and consistent handling to increase measurement reliability. In contrast, mechanical QST in dogs has been reported to be feasible and repeatable under controlled conditions with standardized testing positions (13). Though direct comparison is complicated by differences in species, testing posture, restraint, anatomical compliance, and device used, all of which likely amplify trial-to-trial variability in standing horses. The observed within-session CV of 34%–57% exceeds the 10%–30% typically reported in human studies (27, 28), though it is consistent with previous equine data (7). This likely reflects the inherently binary and unspecific nature of the behavioral endpoint in horses (weight shift, head turning) compared to the verbal self-report used in humans, as well as the greater influence of temperament, distraction, and learned avoidance behavior on threshold determination in horses. Distraction-induced analgesia is well recognized in humans (29), and equine studies have shown that mechanical threshold variability increases after approximately 15 min as horses become bored and more easily distracted (30). In the present study, thresholds were measured at 20-min intervals, which may have contributed to the observed variability. Consequently, it remains uncertain whether the repeatability estimates reported here reflect an inherent characteristic of pressure algometry in horses or a specific consequence of the interval employed in this protocol. The use of a handheld rather than limb-mounted device introduces additional operator-dependent variability in force application, though limb-mounted devices are not readily adaptable to sites beyond the distal limb. The 3-mm probe tip used in this study was selected to deliver focal pressure preferentially activating cutaneous nerve endings, which has been shown to improve measurement consistency compared to larger surface areas (7).

Threshold values recorded in this study were broadly comparable to those previously reported under similar experimental conditions. At the D-MCP joint, our median of 1,495 g aligns with the equivalent of 1,718 ± 332 g reported by Haussler & Hill (31) using a 1-cm probe tip, when accounting for breed differences and probe diameter, though it exceeds the 979 (775–1,244) g reported by Gisler et al. (12) at the same site. At the 7th rib, our values fell within the 495–778 g range previously reported with a 3-mm probe (32). The 7th rib performed poorly overall, which can be explained by the loose overlying skin, underlying muscle mass, and difficulty standardizing tip placement over the bony prominence. At the TMJ, Veres-Nyéki et al. (11) reported a mean of 2,141 g, substantially higher than our values, though it is unclear in that study whether pressure was applied directly over the joint or over the overlying musculature, which would substantially affect the recorded threshold.

Several limitations of this study merit consideration. The modest sample size and biological homogeneity of the cohort (all Thoroughbreds from a single research herd), almost certainly depressed ICC estimates by restricting between-subject variance, limiting generalizability to broader clinical populations. Moreover, entering trials reaching the 55 °C cutoff without eliciting a response biases the variance estimates downward and potentially depressed ICC, as observed values of 55 °C artificially compressed the upper tail of the distribution. However, it must also be acknowledged that nociceptive thresholds may genuinely fluctuate from day to day in a random, biologically real manner, independent of any methodological artefact. The mixed-effects model demonstrating no significant session effect only reveals the absence of systematic directional drift between sessions; it does not capture random scatter around the mean, which is precisely the source of variance to which ICC is sensitive. The present data therefore do not permit us to determine the relative contribution of cohort homogeneity versus true biological variability to the observed poor test–retest reliability. Seasonal and daily temperature fluctuations represent a meaningful source of variability for TT testing, acting through both animal-level factors such as skin temperature, perfusion, and moisture content, and direct interference with device performance. Although testing conditions were broadly similar across sessions, one recording day was markedly colder than the others. The distal limb and the coronary band in particular, rapidly equilibrates to ambient and floor temperature through direct conduction via the bulb and the hoof, producing lower baseline skin temperatures that complicate threshold interpretation. This is in accordance with previous studies, in which the coronary band recorded the lowest threshold temperature in lower environmental temperatures (10). The relationship between environmental temperature and nociceptive threshold is complex and the literature reports conflicting results across species. In cows, when the initial recorded skin temperatures were low, the temperature at which the nociceptive response occurred was higher (33). Similar results were found in horses at the nostrils and withers but not the coronary band (10). To partially address this, sites with skin temperature below 25 °C were pre-warmed to 30 °C–32 °C prior to testing, and the thermode baseline was standardized at 32 °C, consistent with the DFNS protocol used in human QST research (34). Expressing results as thermal excursion percentage (TE%), defined as (threshold temperature − skin temperature) / (cutoff temperature − skin temperature) × 100, has been proposed as a normalization strategy (35), but does not fully resolve the confounding effect of reduced peripheral nerve conduction velocity at lower tissue temperatures, which alters the biological response threshold itself rather than merely shifting the absolute temperature scale. Lower ambient temperature also affects device function. In one study, low ambient and skin temperatures substantially increased the time required to reach the target temperature, reducing the effective power output of the thermal threshold device (10). This was not observed with the device used in the present study. Since the best approach to overcome variations in ambient (and consequentially body surface) temperature has not been established, researchers should aim to keep stable experimental conditions whenever possible, maintaining room temperature around 20 °C. Finally, the heating ramp was set at 0.6 °C/s based on prior equine work demonstrating that reducing the ramp from 0.85 °C/s to 0.5 °C/s yielded clearer behavioral endpoints and more consistent threshold temperatures, though some horses became restless at the lowest rate or skin lesions were more commonly observed (9, 10). Finally, while the operator aimed for a consistent PPT ramp of 200 g/s, the absence of a real-time ramp monitor represents a source of operator-dependent variability that future studies should address through use of devices with integrated force rate feedback.

Collectively, these comparisons suggest that equine QST reliability is shaped by site selection, behavioral responsiveness, and environmental/handling conditions, and that low ICCs, particularly in homogeneous cohorts, may reflect limited between-horse variance as much as measurement error (8, 11, 13, 20).

## 5 Conclusion

In healthy Thoroughbred horses, TT testing demonstrated moderate to good within-session repeatability but poor between-session reliability across all sites, with the coronary band showing particular susceptibility to ambient temperature confounding. Among PPT sites, the D-MCP joint showed the strongest test–retest performance. The absence of systematic change in thresholds between sessions reflects desirable measurement stability; however, poor to moderate ICC values are likely driven in part by the biological homogeneity of this research cohort, which restricts between-subject variance. Averaging repeated trials, strict ambient temperature control, and consistent operator technique are recommended to optimize measurement precision in future equine QST applications. Generalizability of these results to other equine breeds is yet to be established.

## Data Availability

The raw data supporting the conclusions of this article will be made available by the authors, without undue reservation.

## Footnotes

1
randomizer.org
